# High manifestations of food insecurity and hunger among pregnant and lactating women during post-conflict in Tigray, Ethiopia: a community-based study

**DOI:** 10.1186/s12884-024-06550-8

**Published:** 2024-05-24

**Authors:** Ataklti Gebretsadik Woldegebriel, Gebremedhin Gebreegziabiher Gebrehiwot, Ataklti Gessesse Teka, Hagos Amare Gebreyesus, Tesfay Teklemariam Weldeslassie, Abraham Aregay Desta, Haftamu Kebede Yigzaw, Nega Mamo Bezabih, Haylom Kahsay Baryau, Mekonnen Haileselassie, Mengish Bahresilase Gebregziabiher

**Affiliations:** 1 Tigray Health Research Institute, Mekelle, Ethiopia; 2 Adigrate University, Adigrat, Ethiopia; 3 Mekelle University, Adigrat, Ethiopia

**Keywords:** Armed conflict, Food insecurity, Household food insecurity, Hunger Tigray, Ethiopia

## Abstract

**Background:**

Food insecurity is a state or condition in which people have limited or uncertain physical, social, and economic access to safe, sufficient, and nutritious food to meet their dietary needs. Since no thorough evaluation was carried out to determine the degree of household food insecurity in Tigrayan communities in the aftermath of the conflict. This study aims to describe household-level food insecurity status among pregnant and lactating women during the post-armed conflict in Tigray, Ethiopia.

**Method:**

Descriptive research was designed to assess household food insecurity. A multi-stage sampling technique was used for this study. One thousand two hundred forty-nine households were selected systematically following a list of food insecure households. Descriptive statistical values, including frequency counts, percentages, minimum values, maximum values, and averages, were calculated to quantify the indicators under study. Household food insecurity and hunger Scale measurement using the standardized Food and Agriculture Organization standard.

**Results:**

The mean age (± SD) of the mothers was 28.35 ± 5.91 years. More than three fourth of the participants 1010(80.93%) were rural residents. The survey result showed that 88.8% of the pregnant and lactating were food insecure. Half (50.1%) of the households were hungry,one month before the study, 78.5% of the families expressed concern about running out of food and 6.4% had severe hunger.

**Conclusions:**

The food insecurity levels and hunger prestige of the study communities were excessively high. This is in the context of a region affected by intense armed conflict. It is commended that the study communities need to be safeguarded from the direct and long-term consequences of armed conflict-caused household food insecurity.

**Supplementary Information:**

The online version contains supplementary material available at 10.1186/s12884-024-06550-8.

## Introduction

A state or situation known as "food insecurity" occurs when a person's physical, social, or economic access to sufficient, appropriate, and safe food to match their dietary needs or preferences for a productive, healthy, and active life is restricted or unclear [1]. Hunger and its accompanying disorders claim nine million lives annually worldwide, mostly in developing countries [2].

There is a close connection between violent conflict and food insecurity [3]. Food insecurity is a common consequence of conflict, and food insecurity and related social tensions are linked to a higher risk of conflict outbreaks [4]. Put differently, not only does food insecurity result from war, but food insecurity and malnutrition can also serve as catalysts for conflict and serve as conduits for other complaints in situations where there has been a conflict [4, 5].

Relapses into violence are 40% more common in post-war nations with high levels of food insecurity than in those with low levels, according to dependable data from the World Food and Agriculture Organization (FAO) [6]. In general, food shortages and violence impede development efforts by causing physical infrastructure damage, upsetting markets and trade, and taking financial and human resources away from productive industries [7].

From Tigray's perspective, there were several ways in which the recent armed conflict and the tight siege that accompanied it threatened regional food security. In addition to claiming the lives of useful civilians, the battle severely damaged agricultural inputs and infrastructure, including seeds, fertilizer, pesticides, herbicides, and other chemicals [8]. In general, the inhabitants of Tigray have experienced intense conflicts that have impeded the security of food and nutrition as well as the economic development of the region [9]. 89% of households experienced food insecurity at the end of 2022, with 47% experiencing a food crisis or worse, according to the WFP's most recent proxy poll [10].

Nevertheless, food security and nutrition conditions in the region have not improved, if anything they have gotten worse, nine months after the Pretoria peace treaty was signed. Humanitarian help has taken longer to resume. The rising demand has not been met by the available supply. A tiny percentage of persons in need were receiving emergency humanitarian relief, according to the United Nations Office for the Coordination of Humanitarian Affairs (OCHA).

Moreover, there were continuous difficulties in distributing the food assistance once it got to the area. Currently, there is a complete shutdown of the supply and distribution of even more terrible food due to claims of significant diversion of humanitarian goods. Furthermore, the unpaid status of tens of thousands of federal personnel and pensioners has caused them to continue to live in difficulty.

When combined, the aforementioned and other relevant factors are anticipated to worsen the food and nutrition crises in the region. Pregnancy and lactation households' food insecurity levels require accurate and up-to-date data to plan, allocate resources, and target recipients. But when the peace agreement was signed, no thorough evaluation was carried out. To determine the degree of household food insecurity in Tigrayan communities in the aftermath of the conflict, the current study was designed.

## Methodology

**Study setting:** This study was carried out in Tigray regional state, Ethiopia. The survey was conducted from June 24, 2023, to July 25, 2023, **Study design:** A community-based cross-sectional study using a quantitative research approach. The study population was all pregnant or lactating women in the Tigray region. The study unit was every home with a pregnant or lactating woman in the selected Tabias or Kebelles.

### Sampling frame

Health extension workers (HEWs) and partners operating in the selected districts provided the list of lactating and pregnant women from the health posts (HP) of the selected tibias and kebeles of the woreda, which served as the sampling frame for the households.

### Sample size determination

The sample was selected from randomly selected districts to participate in the study. The sample size was calculated using the single population proportion formula assuming a 55% (1) proportion of acute malnutrition among PLW, 5% margin of error, 10% non-response rate, 95% confidence interval, and 3 design effects.

Where:

*N* = expected sample size;

α = 0.05; the probability of detecting a minimum of 5% difference committed by chance.

*P* = 55%; which is the GAM rate among PLW.

q = 1 – p = 45%

DE = 3; design effect.

Therefore,

*N* = ((1.96)2 × 0.55 x (1–0.55)/ (0.05)2)*3 = 1,141; adding 10% non-response (1,141 * 10% = 114 households) with PLW, 1,255 was the total sample size for the survey. Of the 1,255 total samples, at least 30 households with PLW were selected from each Tabia/Kebelle (Table 1).

**Table 1:** Sample distribution for the survey, 2023

Zone	District	Total Population	Total number of Tabias or kebelles	Selected Tabias or kebelles (20%)	Sample size
South Eastern	Hintallo (rural)	109,359	14	3	234
	Samre (urban)	73,248	2	2	136
Central	Abi-Adi (urban)	33,976	2	1	73
	Kolla Temben(rural)	85,896	16	4	181
	Adwa (rural)	118,060	16	4	247
	Adet(rural)	95,550	16	3	200
	Tahtay Maychew(rural)	85,935	12	3	178
Total				19	1249

### Sampling technique and procedure

A multi-stage sampling technique was used for this study. From the seven zones of the region, two zones (Central and South Eastern zones) were chosen using a straightforward random selection method (lottery system). A basic random selection technique was used to choose two districts (Hintallo and Samre) and five (Abi-Adi, Kolla Temben, Rural Adwa, Tahtay Maychew, and Adet) districts from the South Eastern and Central zones, respectively. Before data collection, a minimum of 20% of Tobias were chosen from each district using a basic random selection technique. From each cluster/Tabia, at least 30 PLW with a minimum of one kid between the ages of 6 and 59 months were included. For sampling, a list of homes containing a pregnant and nursing mother was acquired from the field office of the relevant partner or health extension workers (Table 1).

### Data collection tools and procedure

A standardized tool was used to collect the data. The questions were arranged into socio-demographic, obstetric, Food insecurity characteristics, and others. A standardized paper questionnaire was used to collect data from research participants. After extensive revision, the final version of the English questionnaire will be developed. An individual who has fluency in both English and Tigrigna languages will translate the English version to Tigrigna and another individual of similar ability will then back translate the agreed Tigrigna version of the questionnaire to English to check for any inconsistencies or distortion in the meaning of words. Also, it was pretested in 5% of the study population. The data was gathered using an electronic data collecting tool (ODK).

### Data collection

Quantitative information was collected from PLW at selected HHs in two zones and seven districts using a standardized questionnaire. Participants in the study were interviewed at a preselected home. One of the components of the structured quantitative questionnaire was the socio-demographic parameters and demographic traits, along with household food security.

### Food insecurity measurement

Every question had a four-week (30-day) memory period. First was the incidence question, which asked the respondent if the condition had happened at all during the preceding four weeks. When a respondent answered "yes" to the occurrence question, the next question was asked to find out how often the condition had occurred over the preceding four weeks: seldom (one or twice), sporadically (three to ten times), or frequently (more than ten times). The numbers 0 and 1, respectively, were assigned to the "no" and "yes" responses. The totals for the three categories—rarely, occasionally, and frequently—were 1, 2, and 3, respectively. Consequently, the frequency of occurrence score of a particular family on the nine HFIAS questions may vary.

### Household food insecurity experience scale (HFIES) Measures

There are eight questions on the household food insecurity experience scale; one is not included in the HFIAS. There are no questions regarding the frequency of occurrence. The score for the HFIES ranges from 0 to 8. While the household with the higher score has significant levels of food insecurity, the lower-scoring home has little to no food insecurity. Families that scored zero had no food insecurity, three to seven indicated mild food insecurity, six to seven indicated moderate food insecurity, and seven to eight indicated extreme food insecurity (hunger) [5, 6].

### Household food insecurity access scale score

$$\text{AverageHFIASScore}=\frac{\mathrm{Sum}\;\mathrm{of}\;\mathrm{HFIAS}\;\mathrm{scores}\;\mathrm{in}\;\mathrm{thesample}}{\mathrm{Number}\;\mathrm{of}\;\mathrm{HFIAS}\;\mathrm{scores}\left(\text{i}.\text{eHouseholds}\right)\text{inthesample}}$$

### Household Hunger Scale (HHS) measures

The Household Hunger Scale consists of the final three questions (Q7, Q8, and Q9) out of the nine HFIAS questions. The frequency-of-occurrence questions were used to assess the amount of hunger in the questioned households. HHS has a total score range of 0 to 6. Using scores of 0–1, 2–3, and 4–6, respectively, households were classified as having little or no hunger, moderate hunger, or severe hunger [5].

### Data quality assurance

Achieving and sustaining data quality involved aspects such as supervision, training, and the gathering of pilot data before real data collocation.

### Data analysis

Every day, supervisors verified that the data were complete. Data encoders then input the data into SPSS, and the research team used SPSS software version 24 to clean and analyze the data. To quantify the indicators, descriptive statistical values were computed and then evaluated and displayed using tables and bar graphs. These values included frequency counts, percentages, minimum values, maximum values, and averages.

## Results

### Socio- demographiccharacteristics of mothers (1249)

The study comprised 1249 community-dwelling breastfeeding and pregnant women (response rate: 99.5%). The women's mean age (± SD) was 28.35 ± 5.91 years. There were 4.46 households on average. Eighty-nine percent (80.93%) of the participants in the study lived in rural areas. Out of all the women examined, 288 (23.08%) had an educational status and were unable to read and write, and more than half of them, 662 (53.04%), were farmers by occupation (Table 2).

**Table 2:** Socio demographic characteristics lactating and pregnant mothers

Variable	Category	Frequency	Percentage
	Divorced	54	4.33
	Widowed	9	0.72
	Separated	64	5.13
	Other	5	0.40
Age at marriage in years	Mean, SD	18.71(+—3.18)	
Educational status of the participant	Unable to read and write	288	23.08
	Able to read and write	53	4.25
	Elementary	420	33.65
	Secondary	351	28.13
	College and above	136	10.90
Educational status of the head of the household	Unable to read and write	253	20.27
	Able to read and write	199	15.95
	Elementary	356	28.53
	Secondary	276	22.12
	College and above	164	13.14
Religion of the respondent	Orthodox	1219	97.68
	Muslim	29	2.32
Occupation of the participant	Farmer	662	53.04
	Self-employed	152	12.18
	Merchant	114	9.13
	Governmental employed	85	6.41
	Daily laborer	80	6.41
	House wife	155	12.42
Occupation of the head of household	Farmer	703	56.33
	Self-employed	147	11.78
	Merchant	110	8.81
	Governmental employed	134	10.47
	Daily laborer	110	8.81
	House wife	44	3.53
Residence	Rural	1010	80.93
	Urban	238	19.07
Family size	Observations	4.64	1(Min)

### Obstetric characteristics of pregnant and lactating mothers (1249)

Of the 1249 research participants in total, 753 (60.28) were breastfeeding mothers, and 496 (39.82%) were pregnant women. In terms of the frequency of ANC follow-up, there were 115(9.21%), 161(12.9%), 81(6.49%), 53(4.25%), and 34(2.72%) times when there were 4 and above (Table 3).

**Table 3:** Obstatric characterstics pregnant and lactating mothers

Variable	Category	Frequency	Percentage
Are you pregnant	Yes	444	35.58
	No	804	64.42
Mean gestational age	5.6 month	2 month(Minimum)	9 month(Max)
Are you lactating	Yes	778	62.34
	No	470	37.66
ANC Follow up	0	115	9.21
	1	161	12.9
	2	81	6.49
	4	53	4.25
	4 and above	34	2.72
Did you visit health facility for PNC	Yes	639	Yes
	No	609	No

### II Four types of indicators can be calculated

1. Household food insecurity access-related conditions
2. Household food insecurity access-related domains
3. Household food insecurity access scale score
4. Household food insecurity experience scale
5. Household hunger scale
6. Household food insecurity access prevalence

### Household food insecurity access-related conditions

One month before the study, over three-quarters (78.5%) of the families expressed concern about running out of food.In a similar vein, over two-thirds of the households did not have enough money to eat the foods they desired. A limited variety of foods and some items that they did not want to eat are required to be eaten by more than three-fourths (78.8%) and approximately one-third (34.6%) of households, respectively.

In more than half of the families (57.6%), there is never any food of any type available.In the four weeks before the study, at least some members of more than half (52.4%) of the families experiencednighttime hunger. At least some members of the household fast for the entire day and night in around one-third (30.3%) of the homes (Table 4).

**Table 4:** Household food insecurity, Tigray region, 2023 (*n* = 1249)

**1.Access-related conditions**				
**Occurrence questions** **(in the past four weeks)**	**Yes**		**No**	
	**Frequency**	**Percent**	**Frequency**	**Percent**
Did you worry that your household would not have enough food?	981	78.5%	268	21.5%
Were you or any household member not able to eat the kinds of foods you preferred because of a lack of resources?	855	68.5%	394	31.5%
Did you or any household member have to eat a limited variety of foods due to a lack of resources?	984	78.8%	265	21.2%
Did you or any household member have to eat some foods that you really did not want to eat because of a lack of resources to obtain other types of food?	432	34.6%	817	65.4%
Did you or any household member have to eat a smaller meal than you felt you needed because there was not enough food?	984	78.8%	265	21.2%
Did you or any other household member have to eat fewer meals in a day because there was not enough food?	883	70.7%	366	29.3%
Was there ever no food to eat of any kind in your household because of lack of resources to get food?	719	57.6%	530	42.4%
Did you or any household member go to sleep at night hungry because there was not enough food?	654	52.4%	595	47.6%
Did you or any household member go a whole day and night without eating anything because there was not enough food?	379	30.3%	870	69.7%
**2.Domains of food insecurity**				
	Yes		No	
	Frequency	Percent	Frequency	Percent
Anxiety and uncertainty about the household food supply	981	78.5%	268	21.5%
Insufficient Quality (includes variety and preferences of the type of food)	2271	60.6%	1476	39.4%
Insufficient food intake and its physical consequences	3619	58.0%	2626	42.0%
**3.HFI experience**	South Eastern	Central	Overall	
No food insecurity	13.8%	10.1%	11.2%	
Mild food insecurity	21.1%	11.8%	14.6%	
Moderate food insecurity	38.1%	29.4%	31.9%	
Severe food insecurity	27%	48.7%	42.3%	
4.Household hunger scale (HHS)				
Little or no hunger in the HH	64.3%	43.8%	49.9%	
Moderate hunger in the HH	30.3%	49.4%	43.7%	
Severe hunger in the HH	5.4%	6.8%	6.4%	
**5.HFIA prevalence**				
Food secure	15.4%	10.5%	11.9%	
Mildly food insecure	4.9%	3.3%	3.8%	
Moderately food insecure	24.9%	14.3%	66.9%	
Severely food insecure	54.9%	71.9%	88.1%	

### Household food insecurity access-related domains

Food insecurity (access) can be divided into three categories: inadequate quality (including food variety and preferences), anxiety and uncertainty about the household food supply (Question 1), and insufficient food intake and its physical effects (Questions 5–9). Concerns about the availability of food in the home are present in more than three-quarters of the households. Similarly, in the month preceding the study, three out of five households (60.6%) reported inadequate food quality, including preferences for food types and diversity. Inadequate food intake and associated physical effects have affected about 58.0% of the households (Table 4).

### Household food insecurity access scale score

Mean (SD) = 10.76 (6.81).

Median = 11.0

### Household food insecurity experience scale

According to the household food insecurity experience scale, about one in ten households (11.2%) do not have any experience with food insecurity; this percentage was marginally higher in the south-eastern zone (13.8%) than in the central zone (10.1%). In the southeastern zone, the percentage of households experiencing mild to moderate food insecurity was approximately 14.6%, whereas in the central zone, it was substantially higher at 31.9%. Severe household food insecurity has affected over two out of every five households (42.3%), with nearly half (48.7%) in the central zone and more than one-fourth (27.0%) in the southeastern zone (Table 4).

### Household Hunger Scale (HHS)

Only half (49.9%) of the households have little or no hunger. More than two in five (43.7%) and 6.4% of households have moderate and severe hunger, respectively. Around one-third (30.3%) and half (49.4%) in southeastern and central zones, respectively have moderate hunger. Severe hunger was slightly higher in the central zone (6.8%) compared to the southeastern zone (5.4%) (Table 4).

### Household food insecurity access prevalence

In Tigray, just about one in ten homes (11.9%) had enough food, with the percentage significantly higher in the southeastern region (15.4%) than in the central zone (10.5%). 3.8% and 17.5% of households, respectively, experienced mild to moderate food insecurity. One month before the survey, nearly two-thirds (66.9%) of the households experienced acute food insecurity; this percentage was substantially greater in the central zone (71.9%) than in the southeastern zone (54.9%) in Tigray (*p* < 0.001). One month before the survey, approximately 90% of families (88.1%) experienced food insecurity. This was notably greater in the central zone of Tigray (89.5%) than in the southeastern zone (84.6%) (*p* = 0.014) (Table 4).

The household food insecurity experience scale was as follows: light, moderate, severe, 56.8%, 18.4%, 20.4%, and 4.5%, respectively. Food insecurity rates during the conflict (2021) were 0.1%, mild, moderate, and severe (14.9%), 16.1%, 39.1%, and 29.9%, respectively, and 11.2% in this study (post-war).In terms of food insecurity, this study found that it was neither severe nor mild, at 11.2%, 14.6%, 31.9%, and 42.3% (Table 5). x

**Table 5:** Household food insecurity experience scale and Hunger scale stratified by pre-war and war period, Tigray region, 2023 (*n* = 1249)

**HFI experience scale**	Pre-war 2019	During War- A 2021	During War,Nov2021		During war June,2022	Post war June2023( this study)
No Food insecurity	56.8%	-	14/9%		-	11.3%
Mild Food insecurity	18.4%	-	16.1%		-	14.6%
Moderate Food insecurity	20.4%	-	39.1%		-	31.9%
Severe Food insecurity	4.5%	-	29.9%		-	42.3%
**Hunger scale**						
Little or no hunger	96.7%	-	64.2%		48	49.9%
Moderate Hunger	2.4%	-	21.5%		44	43.7%
Sever Hunger	0.9%	-	14.4%		8%	6.4%
HFIA						
No Food insecurity	58.7%	15.4%		37%	47%	11.9%
Mild Food insecurity	11.6%	3.9%		46%	42%	3.8%
Moderately Food insecurity	23.1%	22.8%		15%	10%	17.5%
Severe Food insecurity	6.6%	57.9%		2%	17.5%	66.9%

Pre-war estimates of household hunger in 2019 were 96.7% for little or no hunger, 2.4% for moderate hunger, and 0.9% for severe hunger. There was little to no hunger in 2021, moderate hunger, and severe hunger in 21.5%, 14.4%, and 64.2% of the population, respectively, during the war; in this study, 11.2%( post-war). Little or no hunger, moderate hunger, and severe hunger were seen in 49.9%, 43.7%, 31.9%, and 6.4% of the study participants (Table 5).

Before 2019, the prevalence of food insecurity in households was as follows: mild, moderate, severe, and none at 58.7%, 11.6%, 23.1%, and 6.6%, respectively. No food insecurity throughout the conflict in 2021; mild, moderate, and severe cases were 15.4%, 3.9%, 22.8%, and 57.7%, respectively. There was no food insecurity in this post-war study; instead, the rates were light, moderate, severe, 11.9%, 3.8%, 17.5%, and 66.9% (Table 5).

## Discussion

Food security and stability are closely related [10]. On the other hand, particularly in the globalized era, armed conflicts can be a significant contributor to food insecurity that affects areas outside of the front lines of war; the recent food crises have highlighted the structural difficulties in preventing food insecurity in conflict environments [11].

In Tigray, Northern Ethiopia, the degree of food insecurity (access and experience) and the state of hunger among households affected by the post-armed conflict are examined. According to the HFIAS scale, this survey indicates that, respectively, 11.2% and 88.8% of the households were food secure and food insecure. According to the HFIES measure, 88.9% of the households experienced food insecurity, while 11.1% of the households reported being food secures.

The findings showed a high prevalence of food insecurity among the households under study, which is consistent with the World Food Program's (WFP) assessment, which found that 89% of households in the IPC report (2010) and 88% of households in a study conducted in Pakdasht, Iran [12], were food insecure, along with other severe food security outcomes of armed conflict [13, 14].

According to the HHS scale, 64.1% of the families had little to no hunger and more than one in three (35.8%) of the households experienced moderate to severe hunger. It was comparable to the FAO report. In Ukraine, acute hunger currently affects 26% of the population [15].

Forty-three percent of these households had extreme food insecurity, and thirty percent of them had at least one member who had gone hungry for the entire day or moved out to sleep in the six months before the interview owing to a lack of food or insufficient funds to buy food.

Due to the armed war, households' economic activities and production were disrupted, which resulted in a high prevalence of food insecurity and related problems. The armed conflict in the study communities may have made household food insecurity worse, as reported by the Bureau of Agriculture and Rural Development of Tigray, which reports that 75% of the livestock were killed or looted, all poultry out-growers were disrupted, 85% of all milk processes were disturbed, and 65% of forage processors became dysfunctional [16].

This study highlights a significant issue with the amount, variety, and/or quantity of food that is unavailable for consumption. Due to a lack of resources, household members have noticed a decline in the variety and quality of their food as well as a decrease in the number of meals they eat. The majority of the households expressed concern that they might not have enough food and wouldn't be able to enjoy their favorite cuisines.

In addition, households were required to eat fewer food types, smaller meals, and stuff they did not want to eat. The severity of the armed conflict led to household food insecurity, as evidenced by the fact that over one-third of the homes went without food for the entire day and night.

The continuous armed conflict was the primary source of the concerning food crisis scenario, especially in homes where livelihoods had been lost. One of the reasons for the high levels of food insecurity may be a lack of physical or financial access to enough food as a result of the armed conflict, the siege, or the interruption of humanitarian aid. Long-term, intense combat drove up food prices, which in turn caused poverty, economic damage, and displacement from the battle, and unmanageably high local food prices.

Food security levels have decreased before the war (88.8%), during the war (85.1%), and prewar (43.2%) notwithstanding the cease-fire that was negotiated in Tigray, Ethiopia, and during the postwar scenario. To improve food access and the brittleness of the food systems in the study communities in Tigray, Northern Ethiopia, unrestricted humanitarian access, income-generating activities, and local price controls can therefore be discussed as short-term majors. During the war, the people used little of the reserved food items, but during the post-war period, the reserved food was exhausted and no food aid was provided to the people.

## Conclusions

The food insecurity and hunger status among pregnant and lactating women in the study community were wide excessively high. The armed conflict has a significant negative effect on food security in the Tigray regional state besides, the socio-economic deprivation. It can lead to long-term negative health implications and chronic malnutrition. It is commendable that the study populations should be protected against the short- and long-term effects of family food poverty brought on by armed conflict and socio-economic deprivation. Governments and non-governmental organizations should give special attention, support, and interventions to improving smallholder farmers that increase food self-sufficiency.Additionally, enhancement in investment in agricultural research; improvement in markets, infrastructures, and institutions; and good macroeconomic policies and political systems.

### Supplementary Information

Supplementary Material 1.

## Data Availability

The authors make it possible to share the data and materials used to develop and interpret the results. Please contact Ataklti Gebretsadik- corresponding.
